# A Molecular Basis for Selective Antagonist Destabilization of Dopamine D_3_ Receptor Quaternary Organization

**DOI:** 10.1038/s41598-017-02249-3

**Published:** 2017-05-18

**Authors:** Sara Marsango, Gianluigi Caltabiano, Mireia Jiménez-Rosés, Mark J. Millan, John D. Pediani, Richard J. Ward, Graeme Milligan

**Affiliations:** 1 Centre for Translational Pharmacology, Institute of Molecular, Cell and Systems Biology, College of Medical, Veterinary and Life Sciences, University of Glasgow, Glasgow, G12 8QQ Scotland UK; 2 Laboratori de Medicina Computacional, Unitat de Bioestadística, Facultat de Medicina, Universitat Autònoma de Barcelona, 08193 Bellaterra, Spain; 3 Institut de Recherches Servier, Centre for Innovation in Neuropsychiatry, 125 Chemin de Ronde, Croissy sur Seine, France 78290

## Abstract

The dopamine D_3_ receptor (D_3_R) is a molecular target for both first-generation and several recently-developed antipsychotic agents. Following stable expression of this mEGFP-tagged receptor, Spatial Intensity Distribution Analysis indicated that a substantial proportion of the receptor was present within dimeric/oligomeric complexes and that increased expression levels of the receptor favored a greater dimer to monomer ratio. Addition of the antipsychotics, spiperone or haloperidol, resulted in re-organization of D_3_R quaternary structure to promote monomerization. This action was dependent on ligand concentration and reversed upon drug washout. By contrast, a number of other antagonists with high affinity at the D_3_R, did not alter the dimer/monomer ratio. Molecular dynamics simulations following docking of each of the ligands into a model of the D_3_R derived from the available atomic level structure, and comparisons to the receptor in the absence of ligand, were undertaken. They showed that, in contrast to the other antagonists, spiperone and haloperidol respectively increased the atomic distance between reference α carbon atoms of transmembrane domains IV and V and I and II, both of which provide key interfaces for D_3_R dimerization. These results offer a molecular explanation for the distinctive ability of spiperone and haloperidol to disrupt D_3_R dimerization.

## Introduction

Dopamine receptors are G protein-coupled receptors (GPCRs) that belong to the class A sub-family^1^. They co-ordinate many functions, including motor control, emotional responsiveness and memory consolidation^1^. Moreover, dysregulation of dopaminergic neuro-transmission is implicated in multiple disorders including Parkinson’s disease and a broad suite of psychotic disorders, including schizophrenia^1–3^.

Although class A GPCRs are encoded by single polypeptides that span the plasma membrane seven times and can certainly function as monomeric species^4^, many of these, including the dopamine receptor subtypes, have been shown to form both homo-^5–10^ and hetero-dimers/oligomers^8, 11–15^ both *in vitro* and *in vivo* and this may have functional and clinical significance. Rather less work in this context has focused on the dopamine D_3_ receptor (D_3_R) subtype, which is enriched in limbic areas of the brain and a target for the treatment of, for example, drug addiction and the cognitive and social deficits of schizophrenia and other psychiatric disorders^16, 17^. Even in early studies the potential for dimeric/oligomeric arrangement of this receptor in rodent brain tissue was highlighted^18^, as well as in more recent studies in transfected cell lines that have focused on the extent and basis of such interactions^2, 8, 19, 20^. By combining molecular modelling, site direct-mutagenesis and homogenous time-resolved Fluorescence Resonance Energy Transfer (htr-FRET) techniques, interfaces that allow such interactions have been defined, resulting in description of homomeric quaternary structures of this receptor that involve two distinct dimeric species, as well as a rhombus-shaped tetramer^20^. Unlike members of the class C GPCR sub-family that function as obligate dimers/oligomers^4^, quaternary complexes of the D_3_R are not generated and maintained by covalent interactions between receptor monomers^20^, indicating that the extent of D_3_R dimerization and/or oligomerization will likely be governed by both receptor expression level, ligand availability, and the intrinsic avidity of these protein-protein interactions. This implies that the observed proportions of receptor monomers, dimers and oligomers may well vary between individual cells and tissues and, furthermore, the binding of distinct ligand chemotypes may selectively alter this if they either differentially regulate receptor expression levels or stabilize distinct states of the receptor. Given roles of segments of the seven transmembrane domains (TMDs) of GPCRs that are located close to the extracellular face in controlling class A receptor dimerization^4, 20, 21^ it is clearly possible that different antagonist/inverse agonist-bound structures of the same GPCR may alter the dimerization potential or propensity of the receptor and, therefore, the steady-state distribution of monomers, dimers and oligomers.

Herein we test this hypothesis using the D_3_R for which high affinity blockers from distinct chemotypes are available. Moreover, as an atomic level structure of the D_3_R bound to the ligand eticlopride is available^22^ and substantial efforts have been made to predict modes of binding of other antagonist ligands^23^, this provided a framework with which to assess the outcomes. To answer such questions, we have employed Spatial Intensity Distribution Analysis (SpIDA)^24–27^ as this technique can be used to assess the steady-state proportion of monomers, dimers/oligomers of a cell surface receptor tagged with an appropriate fluorophore, by interrogation and statistical analysis of Regions of Interest (RoI) within confocal images of cells expressing such a receptor^24–27^. We report three key set of outcomes. Firstly, the proportion of the D_3_R present within dimers/oligomers is increased by enhancing levels of receptor expression by treatment of cells expressing the D_3_R with sodium butyrate. Secondly, exposure to some, but not all, chemotypes of ligand with D_3_R antagonist properties markedly reduces the steady-state proportion of D_3_R within dimeric/oligomeric complexes, and thirdly, application of molecular dynamics simulations indicates selective alterations in the tertiary structure of D_3_R caused by ligands that reduce D_3_R quaternary structure complexity and that these are consistent with previously defined key dimer and tetramer-forming interfaces.

## Results

### SpIDA can distinguish between monomer and dimer forms of hD_3_R-mEGFP

**Figure 1:**
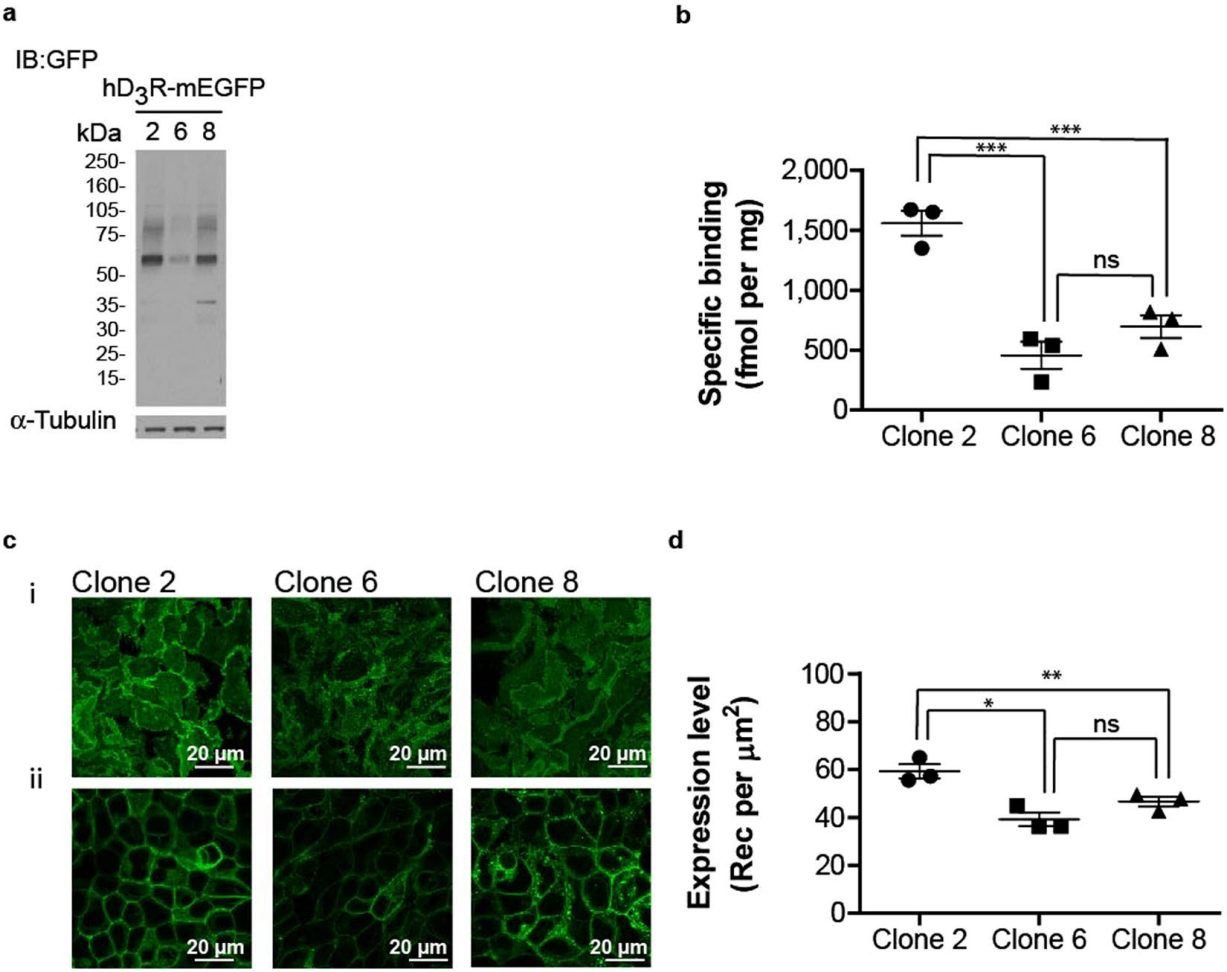
Expression of hD_3_R-mEGFP. hD_3_R-mEGFP was expressed constitutively in Flp-In T-REx 293 cells. Three distinct clones were isolated and characterized. (**a**) Cell lysates were resolved by SDS-PAGE and immunoblotted with an anti-GFP antiserum (*upper panel*) or an anti-α-tubulin antiserum (*lower panel*). (**b**) Specific binding of [^3^H]spiperone (5 nM) was assessed in cell membrane preparations generated from each clone. Data from n = 3 experiments, with the average value from each experiment shown, *error bars* represent ± S.E.M, ***p < 0.001 when compared to the indicated clone, ns = not significant. (**c**) Confocal images were collected from the basolateral membrane **(c(i))** and across the midsection **(c(ii))** of cells. Scale bar = 20 μm. (**d**) Fluorescence intensity analysis of the images akin to those of **(c(i))** allowed estimation of hD_3_R-mEGFP expression levels. Combined data from n = 3 experiments, with the average value from each experiment shown, *error bars* represent ± S.E.M, *p < 0.05; **p < 0.01 when compared to the indicated clone, ns = not significant.

In preparation for studies to assess the steady-state distribution between monomeric and dimeric/oligomeric states of the human (h)D_3_R we expressed constitutively in Flp-In T-REx 293 cells a form of this GPCR tagged at the C-terminus with monomeric, Ala^206^Lys, enhanced green fluorescent protein (mEGFP)^28^. A number of distinct clones expressing this construct were then isolated. Resolution by SDS-PAGE of cell lysates from three clones, followed by immunoblotting with an anti-GFP antiserum, showed a similar pattern in which the predominant form was a polypeptide of some 60 kDa (Fig. 1a). Parallel ligand binding studies using the dopamine D_2_/D_3_ receptor antagonist [^3^H]spiperone provided quantitative validation in that the level of the hD_3_R-mEGFP construct was highest in clone 2 and that there was a trend towards higher levels of the receptor in clone 8 compared to clone 6 (Fig. 1b). This profile was confirmed in the plasma membrane of these cells by observing confocal images (Fig. 1c). Fluorescence intensity analysis of images of the basolateral surface of these clones allowed estimation of expression levels as receptors per μm^2^ (Fig. 1d) that was qualitatively similar to those generated from the specific [^3^H]spiperone binding studies. Overall, these initial studies defined a rank order of expression of the hD_3_R as clone 2 > clone 8 ≥ clone 6 and that the receptor was located predominantly at the cell surface.

**Figure 2:**
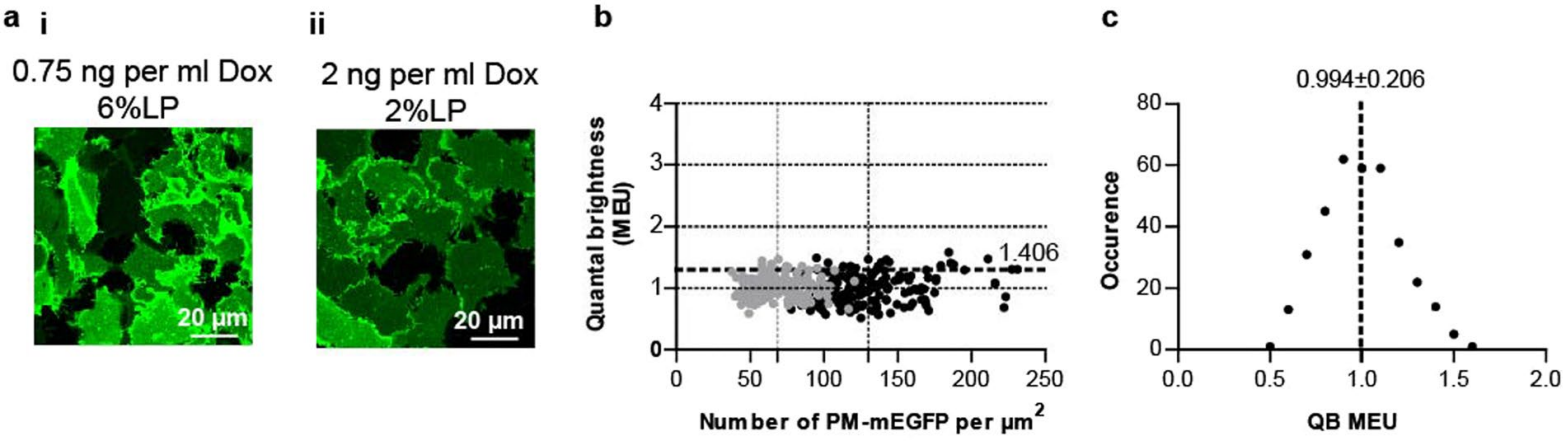
Determination of monomeric QB. Flp-In T-REx 293 cells harboring P + M-mEGFP were treated with 0.75 ng.ml^−1^ or 2 ng.ml^−1^ doxycycline (Dox) for 16 h. (**a**) Confocal images from the basolateral membrane of these cells were collected at 6% **(i)** or 2% **(ii)** laser power respectively. Scale bar = 20 μm. (**b**) QB MEU were assessed in individual RoI and plotted against the number of P + M-mEGFP per µm^2^ at the basolateral surface. *Grey circles*, cells treated with 0.75 ng.ml^−1^ Dox; *black circles* cells treated with 2 ng. ml^−1^ Dox. The horizontal dotted line (1.406 MEU) corresponds to mean + 2 S.D. Values lower or equal to this (95.44% of the data points) were scored as ‘monomeric’ (see ‘Experimental’). Vertical dotted lines provide mean particle number per μm^2^ for the two conditions described above. (**c**) QB MEU values obtained with laser power set at 2% and 6% were combined and binned. Analysis indicated values to be normally distributed.

To be able to define the quaternary structure of hD_3_R within these clones we initially employed control SpIDA^24–27^ studies on Flp-In T-REx 293 cells induced to express differing levels of a single molecule of mEGFP that was targeted to the plasma membrane by N-terminal addition of a palmitoylation + myristoylation (P + M) sequence^27^. Levels of P + M-mEGFP could be varied by addition of different concentrations of doxycycline because the fluorescent protein construct is inserted into a genomic locus from which expression is controlled by this antibiotic in a regulated manner^29^. Using two concentrations of doxycycline that resulted in different levels of P + M-mEGFP expression, SpIDA could be applied effectively on confocal images obtained at 2% laser power only at the higher level of expression. However, at the lower expression level this could also be achieved successfully by increasing laser power to 6% (Fig. 2a). Following treatment with 2 ng.ml^−1^ doxycycline and with laser power set at 2%, analysis of a large number of RoI produced quantal brightness (QB) values for the mEGFP construct of 11.01 ± 0.21 units (mean ± S.E.M., n = 4 × 44, different biological experiments × RoI examined in each experiment) with mean expression level 130 ± 2.5 molecules per μm^2^. As mEGFP is intrinsically monomeric^28^, then in subsequent studies performed at 2% laser power a measured QB for receptor proteins tagged with mEGFP of 11.01 units was defined to represent 1.00 Monomeric Equivalent Units (MEU). Using a similar approach following induction of the cells with 0.75 ng.ml^−1^ doxycycline, and now using 6% laser power, QB of this construct was 24.31 ± 0.31 units (mean ± S.E.M., n = 4 × 44) and when using 6% laser power in subsequent experiments QB = 24.31 was also defined as being equivalent to 1.00 MEU. Here expression levels, 68 ± 1.3 molecules per μm^2^ (mean ± S.E.M., n = 4 × 44), were approximately half of those obtained following induction with the higher concentration of doxycycline (Fig. 2b). Analysis of the full data set showed these to be distributed in Gaussian fashion with MEU of 0.994 ± 0.206 (mean ± S.D., n = 352) (Fig. 2c). This indicates that across the range of expression levels achieved P + M-mEGFP was routinely observed as being monomeric and even at higher levels of expression molecular proximity did not produce artefacts that resulted in this construct being identified incorrectly as being dimeric. The mean + 2 × S.D. corresponded to 1.406 MEU (Fig. 2b) and represents 95.44% of the dataset. In subsequent studies on hD_3_R-mEGFP QB values above 1.406 MEU were thus taken to represent RoI in which dimeric/oligomeric forms were predominant whilst MEU values less than or equal to 1.406 MEU were considered to represent regions in which monomers were the dominant species (see ‘Experimental’ for further details).

**Figure 3:**
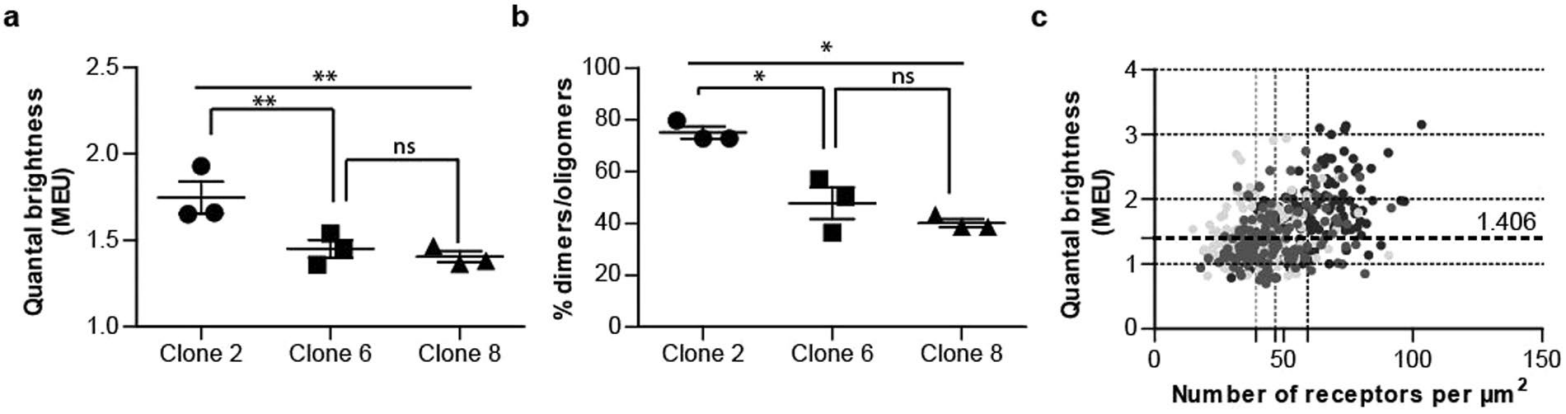
hD_3_R is present as a mixture of complexes: Complexity increases with receptor expression level. (**a**) Overall organizational state of hD_3_R-mEGFP, data is expressed as QB MEU value, with the average value from each experiment shown. Combined data from n = 3 × 44 (experiments × observations), *error bars* represent ± S.E.M. **p < 0.01 when compared to the indicated clone, ns = not significant. (**b**) Assessment of percentage of RoIs containing predominantly dimers/oligomers (characterized by a value of QB MEU > 1.406). Combined data from n = 3 × 44 experiments, *error bars* represent ± S.E.M. *p < 0.05 when compared to the indicated clone, ns = not significant. (**c**) QB MEU were assessed in individual RoI and plotted against the number of hD_3_R-mEGFP per µm^2^ at the basolateral cell surface. *Light grey circles*, cells from clone 6; *dark grey circles* cells from clone 8, *black circles*, cells from clone 2. Horizontal dotted line represents 1.406 MEU, vertical dotted lines provide mean receptor number per μm^2^ for each clone.

Confocal images from the basolateral membrane of cells of clones 2, 6 and 8 were now analysed at 6% laser power. RoIs from each provided fluorescence intensity data on the oligomeric state of hD_3_R-mEGFP. For this specific data set, in clone 2 QB MEU was 1.75 ± 0.09 (mean ± S.E.M., n = 3 × 44) and this was statistically greater than in either clone 6 (1.45 ± 0.05, mean ± S.E.M., n = 3 × 44) or in clone 8 (1.41 ± 0.03, mean ± S.E.M., n = 3 × 44) (Fig. 3a and Supplementary Figure 1). In each clone a substantial number of the individual QB values were > 1.406 MEU, indicating that, at steady-state, in each clone a significant proportion of hD_3_R-mEGFP was identified as being dimeric/oligomeric. Analysis of QB values for each individual RoI indicated that the proportion in which hD_3_R-mEGFP was predominantly within dimeric/oligomeric complexes ranged from 75.0 ± 2.3% (clone 2), to 47.7 ± 6.0% (clone 6) to 40.2 ± 1.5% (clone 8) (Fig. 3b). Interestingly, the assessed higher proportion of hD_3_R-mEGFP present within dimeric/oligomeric complexes within the basolateral membrane of clone 2 correlated with higher expression levels (59.3 ± 1.4 copies per µm^2^, mean ± S.E.M., n = 3 × 44), compared to 49.6 ± 2.1 (clone 8) and 39.2 ± 1.2 (clone 6) copies per µm^2^ (each mean ± S.E.M., n = 3 × 44) at the basolateral surface of cells (Fig. 3c).

### Increasing expression levels of hD_3_R-mEGFP results in a greater extent of receptor dimerization

**Figure 4:**
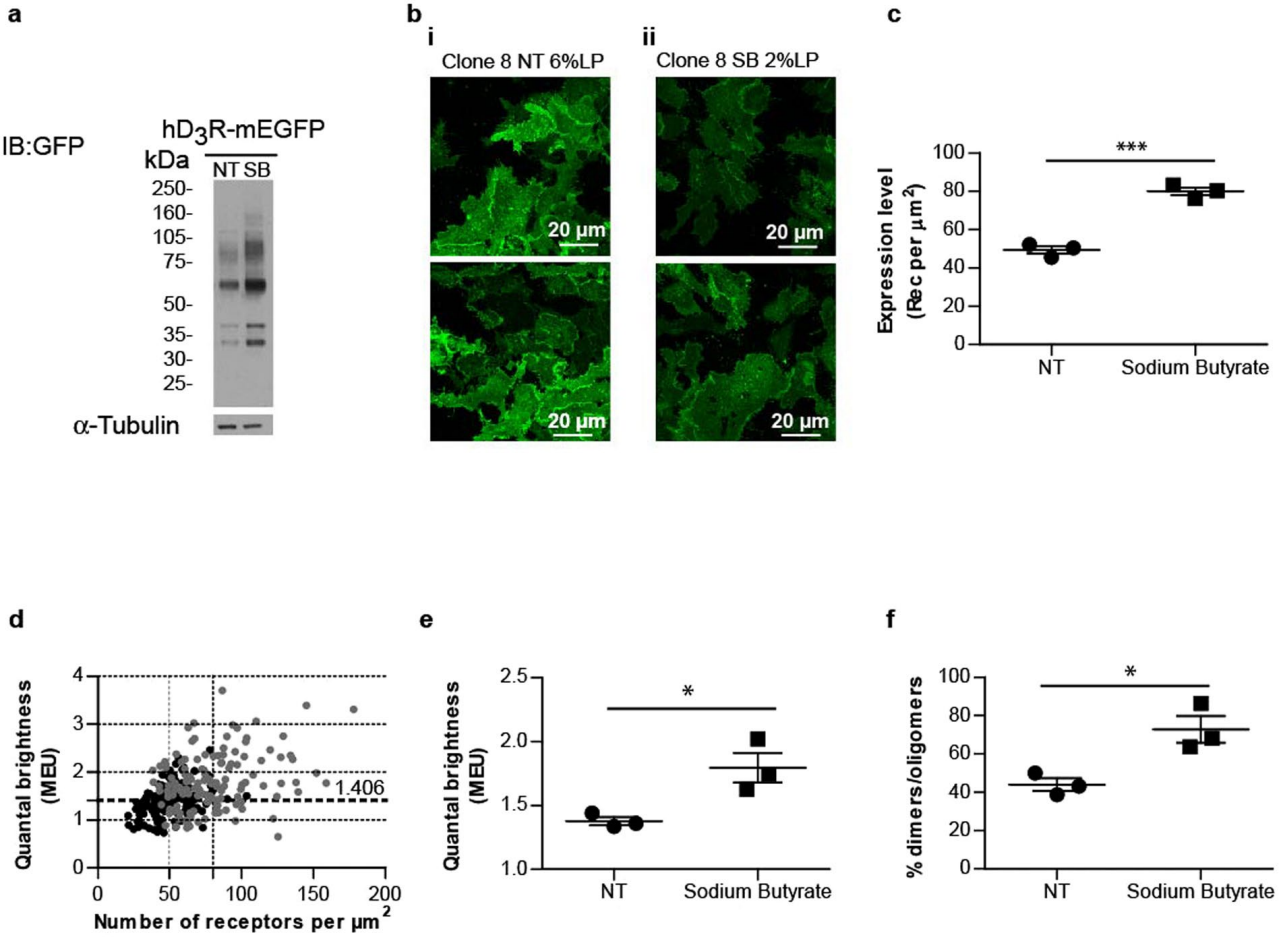
Sodium butyrate increases hD_3_R expression level and oligomeric complexity. Flp-In T-REx 293 hD_3_R-mEGFP clone 8 cells were maintained in the absence (NT) or presence (SB) of 20 mM sodium butyrate for 16 h. (**a**) Lysates from these cells were resolved by SDS-PAGE and immunoblotted with an anti-GFP antiserum (*upper panel*) or an anti-α-tubulin antiserum (*lower panel*). (**b**) Confocal images from the basolateral membrane of these cells were collected with laser power set at 6% (**i**) or 2% (**ii**) respectively. Scale bar = 20 μm. (**c**). Fluorescence intensity analysis of these images indicated hD_3_R-mEGFP cell surface expression level to be higher in cells treated with sodium butyrate. Combined data from n = 3 × 44 (experiments × observations), with the average value from each experiment shown, *error bars* represent ± S.E.M. ***p < 0.001 when compared to not treated. (**d**) QB MEU were assessed in individual RoI and plotted against the number of hD_3_R-mEGFP per µm^2^ at the cell surface. *Black circles*, untreated cells; *grey circles*, cells treated with 20 mM sodium butyrate. Horizontal dotted line (1.406 MEU), vertical dotted lines provide mean receptor number per μm^2^ for not treated and treated. (**e**) Quaternary arrangement of hD_3_R-mEGFP expressed as QB MEU value in not treated cells (1.38 ± 0.03) and cells treated with sodium butyrate (1.80 ± 0.12). Combined data from n = 3 × 44 (experiments × observations), *error bars* represent ± S.E.M., *p < 0.05 when compared to not treated. (**f**) Assessment of percentage of RoIs containing predominantly dimers/oligomers in untreated (43.9 ± 3.3%) and sodium butyrate treated (72.7 ± 6.9%) cells. Combined data from n = 3 × 44 (experiments × observations), *error bars* represent ± S.E.M., *p < 0.05 when compared to not treated cells.

To assess if the proportion of dimeric/oligomeric hD_3_R-mEGFP could be linked more clearly to expression levels of the receptor, rather than clonal variability, we treated cells of clone 8 with the histone deacetylase inhibitor sodium butyrate which has previously been shown to increase cell surface levels of a number of other GPCRs^30, 31^. Overnight treatment with 20 mM sodium butyrate increased levels of hD_3_R-mEGFP as assessed by each of immunoblotting of cell lysates (Fig. 4a), observation of confocal images of the basolateral surface of the cells (Fig. 4b) and assessment of the number of copies of hD_3_R-mEGFP per μm^2^ of the basolateral surface (80.0 ± 2.0 sodium butyrate, 49.4 ± 2.0 untreated, means ± S.E.M., n = 3 × 44) (Fig. 4c). The increase in levels of basolateral membrane receptor produced by treatment with sodium butyrate necessitated subsequent SpIDA being performed on images captured at lower laser power (2%) compared to the untreated cells (6%). When corrected for the difference in QB of mEGFP obtained under the different laser light intensities (see above and Fig. 2 **)**, the assessed MEU value of hD_3_R-mEGFP in the basolateral cell surface was significantly higher in the sodium butyrate-treated cells (Fig. 4d,e) and this corresponded to a higher proportion of dimeric/oligomeric complexes (Fig. 4f).

### Some antagonists of hD_3_R reduce steady-state receptor dimerization

**Figure 5:**
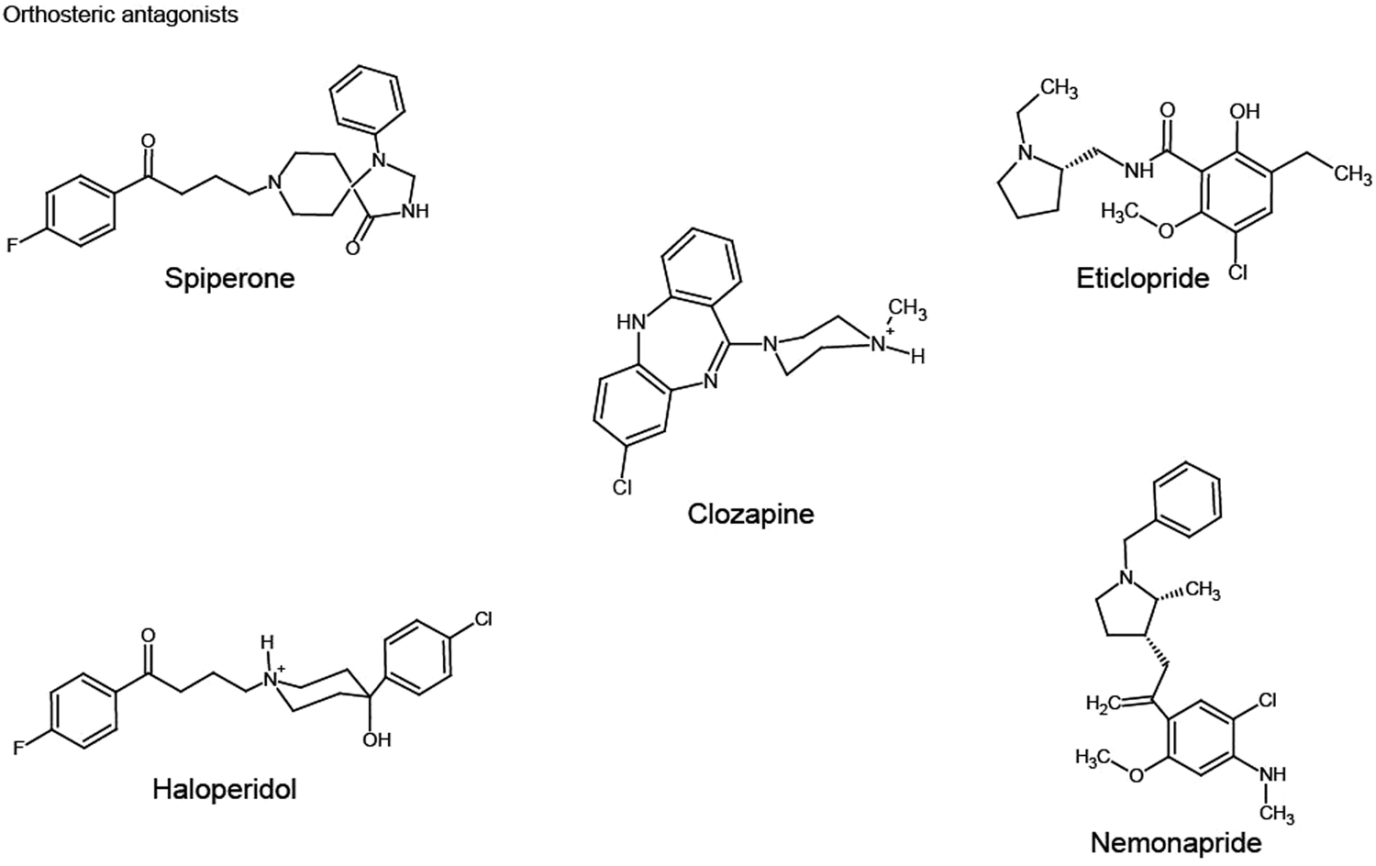
Structure of dopamine receptor antagonists. The chemical structures of the dual D_2_/D_3_ orthosteric antagonists, spiperone, haloperidol, eticlopride, nemonapride and clozapine are shown.

**Figure 6:**
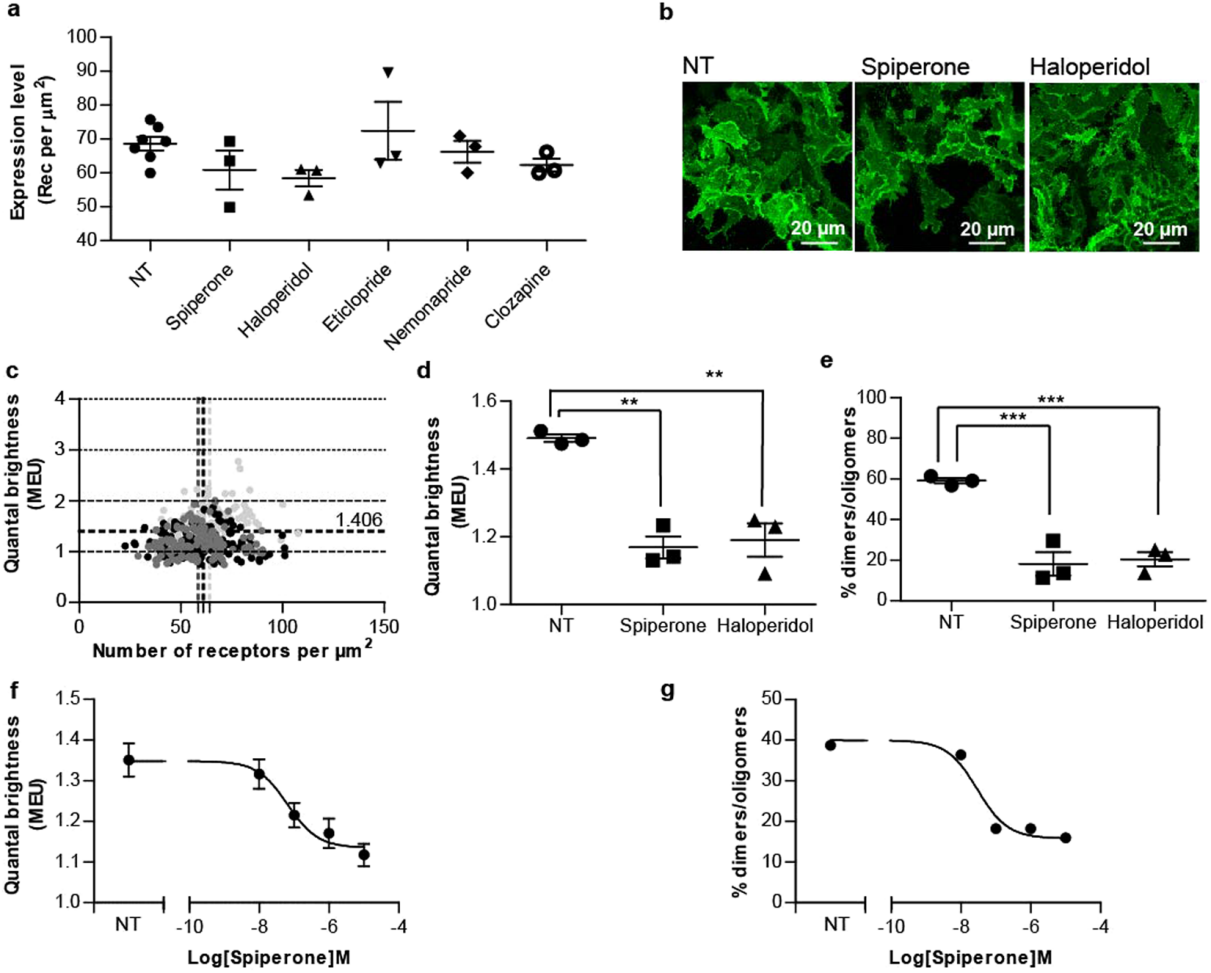
Antagonist treatment does not alter hD_3_R-mEGFP expression levels but spiperone and haloperidol decrease receptor quaternary complexity. Flp-In T-REx 293 hD_3_R-mEGFP clone 2 cells were not treated (NT) or treated with spiperone (10 µM), haloperidol (10 µM), eticlopride (10 µM), nemonapride (10 µM) or clozapine (20 µM) for 16 h. (**a**) Fluorescence intensity analysis of the basolateral membrane of the untreated cells indicated hD_3_R-mEGFP to be expressed at 68.6 ± 2.0 receptor per µm^2^ and that treatment with the various antagonists did not significantly alter cell surface expression level. Combined data from n = 10 × 44 experiments × observations (NT) and 3 × 44 (each antagonist), with the average value from each experiment shown, *error bars* represent ± S.E.M. (**b**) Confocal images from the basolateral membrane of cells not treated (NT) or treated with spiperone or haloperidol were collected with laser power set at 6%. Scale bar = 20 µm. (**c**) QB MEU were assessed in individual RoI and plotted against the number of hD_3_R-mEGFP per µm^2^ at the cell surface. *Light grey circles*, untreated cells; *dark grey circles*, cells treated with haloperidol; *Black circles*, cells treated with spiperone. (**d**) Quaternary arrangement of hD_3_R-mEGFP receptor expressed as QB MEU value in not treated cells (1.49 ± 0.01) and cells treated with spiperone (1.17 ± 0.03) or haloperidol (1.19 ± 0.05). Combined data from n = 3 × 44 measurements, *error bars* represent ± S.E.M. **p < 0.01 when compared to not treated. (**e**) Assessment of percentage of RoIs containing predominantly dimers/oligomers in untreated (59.1 ± 1.3%) or treated with spiperone (18.2 ± 5.7%) or haloperidol (20.5 ± 3.5%). Combined data from n = 3 × 44 experiments, *error bars* represent ± S.E.M., ***p < 0.001 when compared to not treated cells. (**f**) QB MEU mean values were plotted against spiperone concentration. Combined data from n = 44 measurements, *error bars* represent ± S.E.M. (**g**) The percentage of RoI containing predominantly dimers/oligomers was calculated and plotted against spiperone concentration.

We next assessed if ligands from distinct chemical classes that act as antagonists/inverse agonists of the hD_3_R could alter receptor quaternary organization. Treatment of clone 2 cells for 16 hours with concentrations (10 μM) of spiperone and haloperidol (Fig. 5) that were calculated to be at least 100 times their K_d_ at hD_3_R (Table 1) had no effect on overall expression levels of the receptor (Fig. 6a). This was also the case following treatment with other, structurally distinct (Fig. 5), orthosteric antagonist ligands with affinity at the hD_3_R including eticlopride, nemonapride and clozapine (Fig. 6a). Confocal images of the basolateral membrane of control and either spiperone- or haloperidol-treated cells were collected (Fig. 6b) and analysed using SpIDA (Fig. 6c). In haloperidol-treated cells the receptor construct now displayed calculated MEU 1.19 ± 0.05 (mean ± S.E.M., n = 3 × 44) (Fig. 6d) which was markedly lower (p < 0.01) than untreated cells (MEU 1.49 ± 0.01, mean ± S.E.M., n = 3 × 44 in this specific experimental dataset) and analysis of the individual images showed that the receptor was now predominantly monomeric (Fig. 6e). Similar effects were produced by treatment with spiperone (average MEU = 1.17 ± 0.03, mean ± S.E.M, n = 3 × 44) (Fig. 6c–e). Furthermore, the ability of spiperone to favor monomerization of the hD_3_R was concentration-dependent (Fig. 6f,g).

**Table 1:** Affinity of selective antagonists at hD_3_R-mEGFP.

Compound	pKi
Spiperone	8.74 ± 0.08
Haloperidol	7.87 ± 0.15
Eticlopride	10.10 ± 0.26
Nemonapride	9.28 ± 0.49
Clozapine	6.03 ± 0.14

Values were calculated based on the ability of each ligand to compete with [^3^H]spiperone to bind hD_3_R-mEGFP.

**Figure 7:**
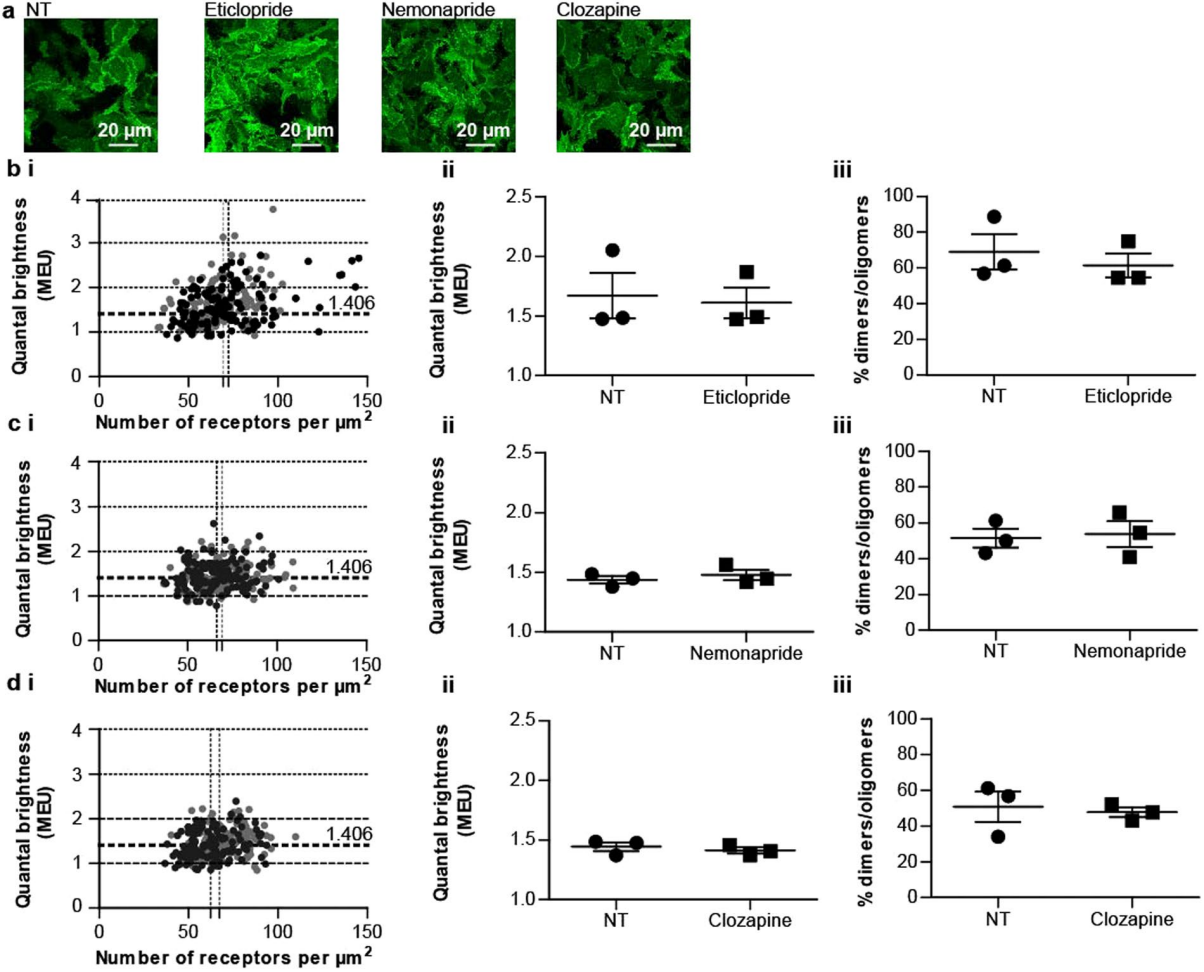
Treatment with eticlopride, nemonapride or clozapine does not alter the quaternary organization of hD_3_R. Flp-In T-REx 293 hD_3_R-mEGFP clone 2 cells were untreated or treated with eticlopride, nemonapride or clozapine as described in Fig. 6. (**a**) Confocal images from the basolateral membrane of cells not treated (NT) or those treated with eticlopride, nemonapride, or clozapine were collected with laser power set at 6%. Scale bar = 20 μm. QB MEU were assessed in individual RoI and plotted against the number of hD_3_R-mEGFP per µm^2^ at the cell surface. *Light grey circles*, untreated cells (NT); *Black circles*, cells treated with eticlopride (**b(i)**), nemonapride **(c(i)**), or clozapine (**d**(**i**)). Quaternary organization of hD_3_R–mEGFP receptor was expressed as QB MEU value and was equal to 1.67 ± 0.19 and 1.61 ± 0.13 in vehicle- and eticlopride- treated cells, respectively (**b**(**ii**)); to 1.44 ± 0.03 and 1.48 ± 0.04 in vehicle- and nemonapride treated cells, respectively (**c**(**ii**)) and to 1.44 ± 0.04 and 1.41 ± 0.02 in vehicle- and clozapine treated cells, respectively (**d**(**ii**)). RoIs containing predominantly dimers/oligomers were found to be equal to 68.9 ± 10.0 and 61.4 ± 6.8 in vehicle- and eticlopride treated cells, respectively (**b**(**iii**)); to 51.5 ± 5.3 and 53.8 ± 7.2 in vehicle- and nemonapride treated cells, respectively (**c**(**iii**)) and to 50.8 ± 8.4 and 47.7 ± 2.6 in vehicle- and clozapine treated cells, respectively (**d**(**iii**)). Combined data each from n = 3 × 44 experiments, with the average value from each experiment shown, *error bars* represent ± S.E.M.

Interestingly, the effects of the butyrophenones spiperone and haloperidol on hD_3_R quaternary structure were not reproduced by other antagonists with high affinity for the hD_3_R. Thus, treatment of cells of clone 2 with the benzamides eticlopride or nemonapride, which are structurally closely related to each other (Fig. 5), but not to either spiperone or haloperidol (Fig. 5), did not alter mean MEU nor in the proportion of RoIs assessed as containing predominantly receptor dimers/oligomers versus monomers (Fig. 7). This was also the case for the atypical antipsychotic and tricyclic benzodiazepine clozapine (Fig. 7).

### Spiperone treatment does not alter the quaternary structure of hD_3_R-Asp^110^Ala-mEGFP receptor

**Figure 8:**
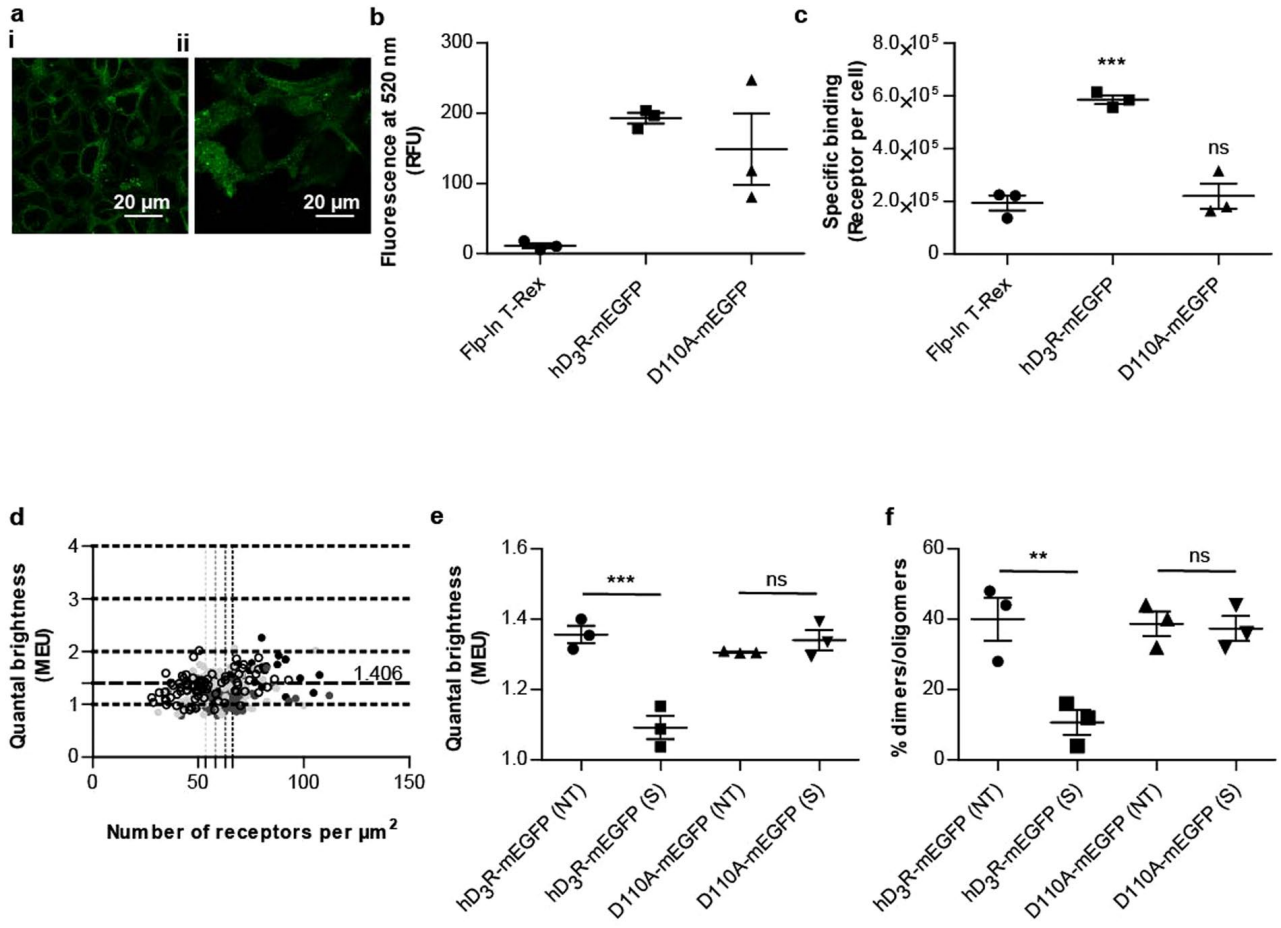
Treatment with spiperone does not alter the quaternary organization of hD_3_R-Asp^110^Ala. hD_3_R-Asp^110^Ala-mEGFP was expressed constitutively in Flp-In T-REx 293 cells. Confocal images were collected across the midsection (**a**(**i**)) and from the basolateral membrane (**a**(**ii**)) of cells. Scale bar = 20 μm. (**b**) Fluorescence intensity corresponding to mEGFP was measured in non-transfected (*circle*), hD_3_R-mEGFP- (*square*) or hD_3_R-Asp^**110**^Ala -mEGFP (*triangle*) expressing Flp-In T-REx 293 cells. Data from n = 3 experiments, with the average value from each experiment shown, *error bars* represent ± S.E.M. (**c**) Specific binding of [^3^H]spiperone (5 nM) was assessed in either non-transfected (*circle*), hD_3_R-mEGFP (*square*) or hD_3_R-Asp^**110**^Ala -mEGFP (*triangle*) expressing Flp-In T-REx 293 cells. Data from n = 3 experiments with the average value from each experiment shown, *error bars* represent ± S.E.M. ns = not significant; ***p < 0.001 when compared to non-transfected Flp-In T-REx 293 cells. (**d**) QB MEU were assessed in individual RoI and plotted against the number of receptors per µm^2^ at the cell surface. *Black circles*, untreated hD_3_R-mEGFP expressing cells; *Dark grey circles*, spiperone treated hD_3_R-mEGFP expressing cells; *Light grey circles*, untreated hD_3_R-Asp^110^Ala-mEGFP expressing cells; *Open circles*, spiperone treated hD_3_R-Asp^**110**^Ala -mEGFP expressing cells. (**e**) Quaternary arrangement of hD_3_R-mEGFP or hD_3_R-Asp^**110**^Ala-mEGFP receptors expressed as QB MEU value in not treated cells ((NT) 1.36 ± 0.02 and 1.31 ± 0.03, respectively) and cells treated with spiperone ((S) 1.09 ± 0.03 and 1.34 ± 0.03, respectively). Combined data from n = 3 × 25 measurements, *error bars* represent ± S.E.M. ns = not significant; ***p < 0.001 when compared to the indicated sample. (**e**) Assessment of percentage of RoIs containing predominantly hD_3_R-mEGFP or hD_3_R-Asp^**110**^Ala -mEGFP dimers/oligomers in untreated ((NT) 40.0 ± 6.1% and 38.7 ± 3.5%, respectively) or treated with spiperone ((S) 10.7 ± 3.5% and 37.3 ± 3.5%, respectively). Combined data from n = 3 × 25 experiments, *error bars* represent ± S.E.M., ns = not significant; **p < 0.01 when compared to the indicated sample.

To assess whether the effect of spiperone in favoring the monomeric state of hD_3_R directly reflected direct binding of the ligand to the receptor or potentially a non-specific effect at the level of the cell membrane, we generated hD_3_R-Asp^110^Ala-mEGFP. Alteration of this aspartate residue is known to greatly reduce the affinity of the receptor for spiperone^32^. Expression of hD_3_R-Asp^110^Ala-mEGFP in Flp-In T-REx 293 cells was demonstrated by confocal microscopy (Fig. 8a) and confirmed by monitoring fluorescence at 520 nm corresponding to mEGFP (Fig. 8b) to be at equivalent levels of expression as hD_3_R-mEGFP (Fig. 8b). Parallel ligand binding experiments confirmed that hD_3_R-Asp^110^Ala-mEGFP was unable to bind [^3^H]spiperone (Fig. 8c). SpIDA of images from RoIs, assessed at 6% laser power, indicated that at steady-state the percentage of RoIs containing mainly dimers /oligomers of hD_3_R-Asp^110^Ala-mEGFP was not different from that observed in cells expressing equal levels of hD_3_R-mEGFP (Fig. 8d–f). Treatment of cells expressing hD_3_R-Asp^110^Ala-mEGFP with spiperone (10 µM) did not, however, alter mean MEU nor the calculated proportion of RoIs containing predominantly receptor dimers/oligomers (Fig. 8d–f).

### The effect of spiperone on hD_3_R monomerization is reversible but cannot be attributed to ligand inverse agonism or ligand-induced differences in receptor mobility

**Figure 9:**
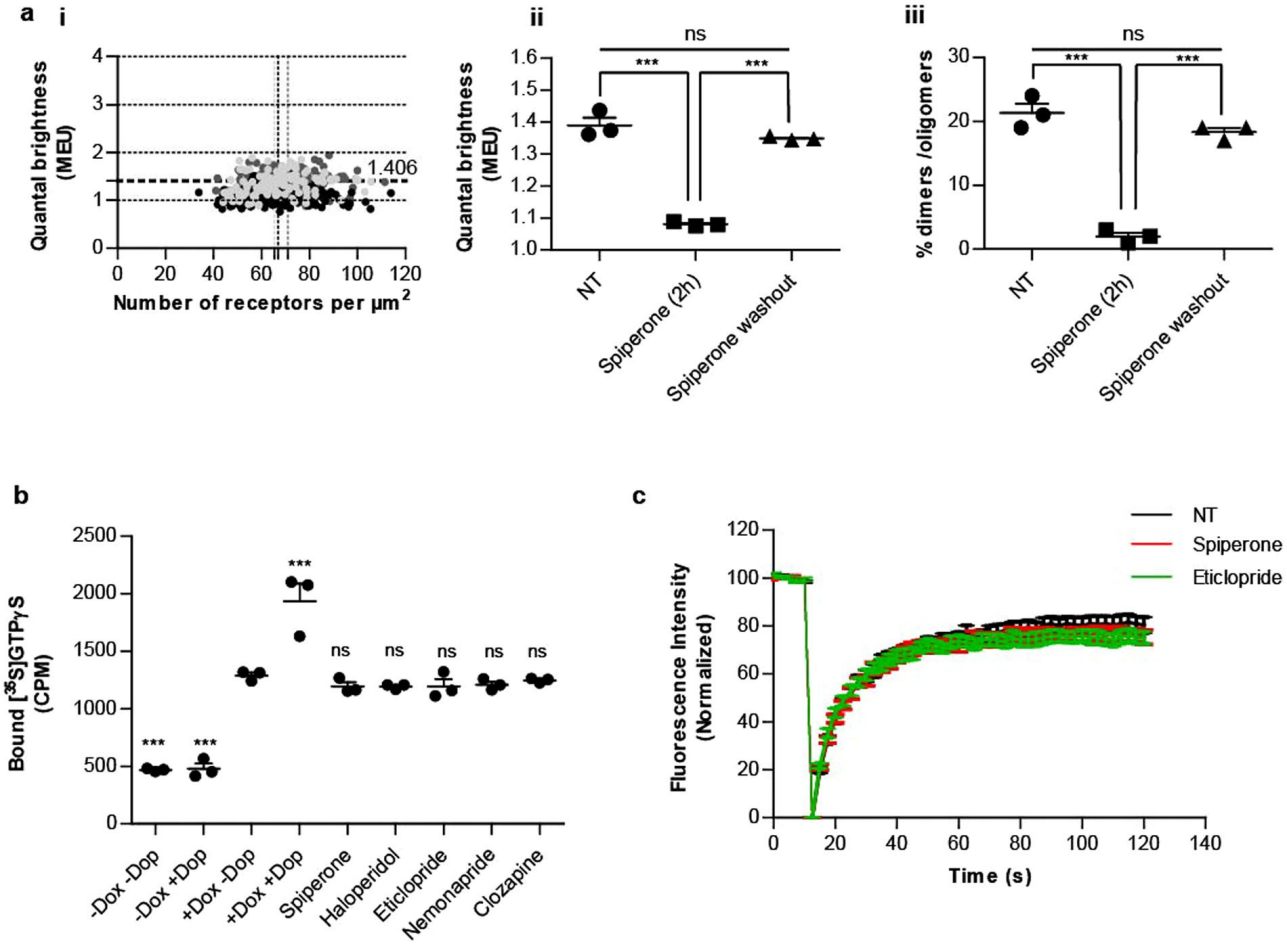
The effect of spiperone on hD_3_R quaternary arrangement is reversible and cannot be attributed to ligand inverse agonism or ligand-induced differences in receptor mobility. (**a**) Flp-In T-REx 293 hD_3_R-mEGFP clone 2 cells were untreated (NT), treated with spiperone for 2 hours (spiperone (2 h)) or treated with spiperone for 2 hours and washed in growth medium at 37 °C for 4 hours (spiperone washout). QB MEU were assessed in individual RoI and plotted against the number of hD_3_R-mEGFP per µm^2^ at the cell surface. *Dark grey circles*, untreated cells (NT); *Black circles*, cells treated with spiperone for 2 h*; Light grey circles*, cells treated with spiperone for 2 h and washed in growth medium for 4 h (**a**(**i**)). Quaternary organization of hD_3_R–mEGFP receptor was expressed as QB MEU value and was equal to 1.39 ± 0.02, 1.08 ± 0.01 and 1.35 ± 0.01 in vehicle-, spiperone- and spiperone-treated followed by washout of the ligand cells, respectively (**a**(**ii**)). RoIs containing predominantly dimers/oligomers corresponded to 21.3 ± 1.5, 2.00 ± 0.6 and 18.3 ± 0.7% in vehicle-, spiperone- and spiperone-treated followed by washout cells, respectively (**a**(**iii**)). Combined data from n = 3 × 44 experiments (performed in parallel), with the average value from each experiment shown, *error bars* represent ± S. E.M., ***p < 0.001, when compared to the indicated treatment, ns = not significant. (**b**) Membranes from Flp-In T-REx 293 hD_3_R + Cys^352^Ile G_o1_ cells maintained in the absence (−Dox) or presence of 100 ng.ml^−1^ doxycycline (+Dox) were used to measure basal [^**35**^S]GTPγS binding (−Dop) and the effect of 10 μM dopamine (+Dop). Addition of receptor saturating concentrations of spiperone, haloperidol, eticlopride, nemonapride or clozapine did not reduce the basal level of [^35^S]GTPγS binding. Combined data from n = 3 experiments, *error bars* represent ± S. E.M., ***p < 0.001, ns = not significant when compared to basal (+Dox −Dop). (**c**) Analysis of the recovery of fluorescence intensity after mEGFP photobleaching at the basolateral membrane of Flp-In T-REx 293 hD_3_R-mEGFP clone 2 cells untreated (black) or treated with receptor saturating concentration of spiperone (red) or eticlopride (green). Combined data from n = 11 experiments, *error bars* represent ± S.E.M.

We next explored whether ligand-induced monomerization of hD_3_R was reversible. Initially the dissociation rate of [^3^H]spiperone from hD_3_R-mEGFP in intact cells at 37 °C was assessed. After allowing binding of [^3^H]spiperone to reach equilibrium, washout of unbound ligand allowed dissociation of [^3^H]spiperone to be followed under conditions of infinite dilution. Dissociation of bound [^3^H]spiperone followed a mono-exponential curve with K_off_ = 0.014 min^−1^ and, therefore, had a half-time in the region of 51 min. After treatment of cells with spiperone for 2 hours to promote receptor monomerization, the bulk drug was washed out and images collected 4 hours later by which point the remaining drug was anticipated to have dissociated from the receptor. Now QB and SpIDA showed that receptor complexity had re-established to reach levels of hD_3_R dimerization that were not statistically different from that of untreated cells (Fig. 9a). Although all the ligands employed are functional antagonists of the D_3_R we explored whether ligands that promoted monomerization could be sub-classified on the extent that they acted as receptor inverse agonists as regards their G-protein coupling. In Flp-In T-REx 293 cells constitutively expressing hD_3_R and harboring the α subunit of a Cys^352^Ile, pertussis toxin-resistant, form of G_o1_^33^, treatment with doxycycline to promote expression of the G protein resulted in enhanced levels of basal [^35^S]GTPγS binding, and this was increased further by addition of 10 μM dopamine (Fig. 9b). Addition of receptor saturating concentrations of each of spiperone, haloperidol, eticlopride, nemonapride or clozapine did not significantly reduce the basal level of [^35^S]GTPγS binding (Fig. 9b), indicating that these five antagonists could not be separated based on varying degrees of inverse agonism. Ligand-induced alterations in receptor mobility could potentially also affect SpIDA. We also employed fluorescence recovery after photobleaching (FRAP) to measure receptor movement, both in the unliganded, basal state and in the presence of receptor saturating concentrations of either spiperone or eticlopride (Fig. 9c). In the unliganded state, recovery of fluorescence corresponding to hD_3_R-mEGFP into a RoI followed a mono-exponential with t_0.5_ = 9.6 ± 0.5 seconds and assessed mobile fraction of 79.4 ± 2.9% (means ± S.E.M., n = 11). In the presence of either spiperone or eticlopride the assessed mobile fraction was unaltered, indicating that drug treatment did not result in variable detection of hD_3_R-mEGFP (Fig. 9c). Moreover, the time constant for recovery was not different between cells treated with either spiperone or eticlopride.

### Ligand docking and molecular dynamics simulations provide a basis for selectivity of ligand effects

**Figure 10:**
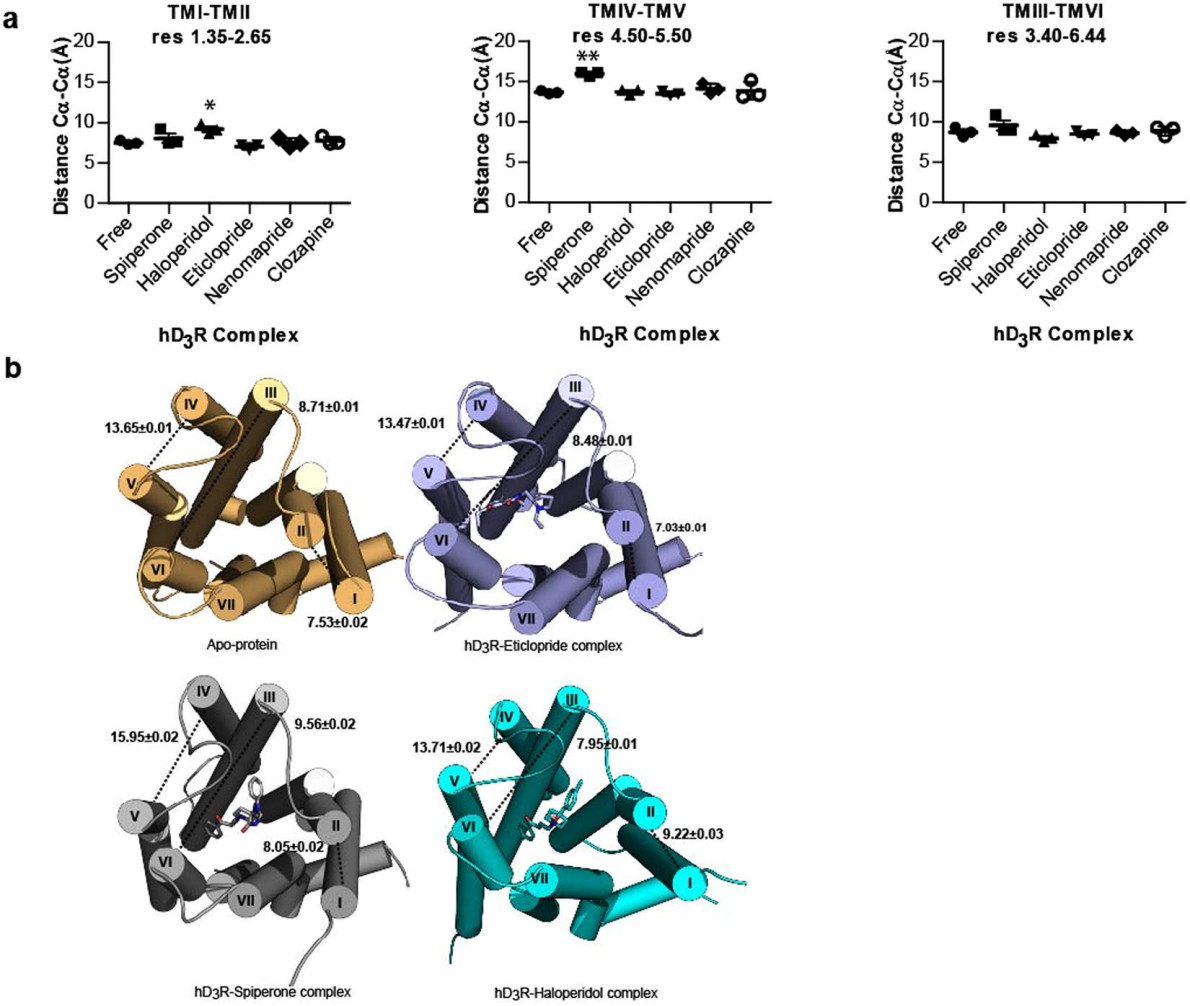
Molecular dynamics simulations indicate that binding of spiperone and haloperidol induces distinct conformational changes in the tertiary structure of hD_3_R. Molecular dynamics simulations of the hD_3_R apo-protein, or hD_3_R bound with eticlopride, spiperone, haloperidol, clozapine or nemonapride were run for 500 ns. (**a**) Comparison of the distances between the residues located at positions 1.35 and 2.65 in TMD I-TMD II, 3.40 and 6.44 in TMD III-TMD VI and 4.50 and 5.50 in TMD IV-TMD V in the hD_3_R is shown. Data are means ± SEM from n = 3 experiments, with the average value from each experiment shown, *p < 0.05; **p < 0.01 when compared with the apo-protein. See Results for details of quantification. (**b**) Representation of the tertiary structure of the receptor at the end of this period. The distances between residues within helices (calculated as distance between the pairs of c-alphas denoted above) are shown.

To provide potential insight into the molecular basis for the distinct effects of spiperone and haloperidol versus eticlopride, nemonapride and clozapine on hD_3_R quaternary organization we turned to the use of molecular dynamics simulations. Eticlopride was of particular interest because an atomic level structure of this ligand bound to the D_3_R is available^22^. We thus compared molecular dynamics simulations of the minimal energy state of hD_3_R bound by each of these ligands (see ‘Experimental’ for further details), alongside the apo-protein, and how their binding might affect the organization of the extracellular side of the TMD helices (Fig. 10 and Supplemental Figure 2). These showed distinct differences in the orientation of the extracellular face of hD_3_R when associated with spiperone and haloperidol compared to when bound by eticlopride, nemonapride or clozapine and also compared to the apo-protein. Analysis of the distances between helices (as measured between defined pairs of amino acid α carbon atoms), indicated that whilst both spiperone and haloperidol produced marked changes, each ligand stabilized a different inactive conformation of the hD_3_R. Spiperone-bound hD_3_R induced a significantly increased distance, by 2.3 Å on average, between TMD IV (residue 4.50) and TMD V (residue 5.50) (residue numbers reflect the positional reference numbering system introduced by Ballesteros and Weinstein)^34^ compared to the apo-protein (p < 0.01), whereas none of the other ligands induced such a change (Fig. 10 and Supplementary Figures 2 and 3). Although not achieving statistical significance, there was also a trend for spiperone to induce an increase in the distance between TMD I (residue 1.35) and TMD II (residue 2.65) and between TMD III (residue 3.40) and TMD VI (residue 6.44). This is interesting since binding of haloperidol induced a statistically significant increase of 1.7 Å (p < 0.05) in distance between TMD I (residue 1.35) and TMDII (residue 2.65). However, haloperidol did not alter the mean distance between residues 4.50 and 5.50. Importantly, binding of eticlopride, nemonapride and clozapine was without effect on distances between these specific residue pairs (Fig. 10 and Supplemental Figure 3).

Interestingly, based on mutagenesis studies, we have previously proposed residues from each of TMD IV-TMD V and TMD I-TMD II helices as generating active interfaces in hD_3_R dimers and tetramers^20^ based on the available eticlopride-bound hD_3_R atomic level structure. The spiperone-induced alteration in the distance between residues of TMD IV and TMD V and haloperidol-induced alteration in the distance between TMD I and TMD II residues, may be integral to why spiperone and haloperidol but not the other ligands produced reduced levels of hD_3_R dimers/oligomers.

## Discussion

The idea that members of the class A sub-family of GPCRs can form dimers and/or oligomers has been hypothesized and tested for a considerable period^4^. Key questions that remain uncertain or unanswered include whether such complexes are stable or transient, if the extent of oligomerization is determined, at least in part, by receptor expression levels, and to what extent the steady-state level of such quaternary organization can by altered by the binding of receptor ligands. Recently, the first two of these issues have begun to be addressed. Using single molecule tracking studies clear evidence has now been provided that interactions between individual monomers, of at least muscarinic acetylcholine receptor subtypes^35, 36^, the dopamine D_2_ receptor^37^ and the N-formyl peptide receptor^38, 39^, are transient. Moreover, consistent with Mass-Action, for both the β_1_- and β_2_-adrenoceptors^40^, the serotonin 5-HT_2C_ receptor^41^, the dopamine D_2_ receptor^37^ and the opsin receptor^42^ quaternary complexity has been reported to increase with higher levels of expression of the receptor. Although this is in contrast to earlier conclusions for both the dopamine D_2_ receptor^5–7^ and the serotonin 5-HT_2c_ receptor^43^ where the reported extent of dimerization remained constant across expression ranges of at least 10 fold, herein we provide further support of the importance of Mass-Action in this process by showing that quaternary complexity of the D_3_R also increases with receptor expression level. To explore this, we took advantage that a number of previous studies had indicated that treatment of cells expressing a GPCR of interest with sodium butyrate can result in up-regulation of receptor levels^30, 31^. Overnight treatment of cells stably expressing D_3_R-mEGFP with sodium butyrate, that resulted in less than a doubling of receptor number, was associated with a marked increase in the proportion of RoIs examined in which dimeric/oligomeric organization of the receptor was dominant. This approach eliminated potential caveats of effects of clonal variability in determining the extent of receptor structural complexity.

In the current experiments hD_3_R was expressed in the HEK293-based cells at levels, based on the specific binding of [^3^H]spiperone, between 500–1500 fmol per mg membrane protein in the three individual clones used. Clone 2 expressed the receptor to the highest levels. Based on surface area of these cells, determined to be 5235 µm^2^ (see ‘Experimental’ for calculation of this value), the amount of membrane protein generated per 10^6^ cells, and the apparently uniform distribution of the receptor within the plasma membrane as seen in cross-sectional confocal images, this yielded a predicted average receptor density as some 81 receptors per µm^2^. This value was both similar to estimates provided within the SpIDA software and, importantly, was centrally located within the range of molecules per μm^2^ of P + M mEGFP used for the calibration studies, in which no appearance of apparent dimers of P + M mEGFP was observed that could have reflected simple molecular proximity due to high levels of protein expression.

One of the major outstanding issues in considering class A GPCR quaternary structure and its implications is the effect, if any, of ligands on receptor organizational structure. This has been a much discussed and debated topic^44–46^ with at least as many reports indicating that ligands have little or no effect than those which argue differently^47, 48^. In the current studies, we add to and extend this topic greatly. Firstly, we show that certain antagonists with high affinity for the D_3_R strongly favor reduction in quaternary complexity of this receptor and promote its monomerization. Such an effect may provide a rational for why Tabor *et al*.^37^, reported very modest levels of D_3_R dimerization as the analysis was based on the binding of a fluorescent antagonist to the receptor. Ligands that promoted monomerization of the D_3_R comprised the chemically-related butyrophenones, spiperone and haloperidol. However, this was not a universal effect produced by binding of any antagonist with high affinity for the D_3_R. Interestingly, among ligands lacking this effect was the benzamide eticlopride, the ligand used to enhance stabilization and crystallization of the D_3_R in the studies of Chien *et al*.^22^. Perhaps not surprisingly in this regard given its chemical relatedness to eticlopride, nemonapride also failed to alter the balance between D_3_R monomers and dimers/oligomers, and this was also the case for the tricyclic dibenzodiazepine and atypical antipsychotic drug clozapine.

We have previously noted that certain antagonists of the 5-HT_2C_ receptor also favor monomerization of that receptor, but in those studies we did not identify antagonist ligands that did not have such an effect^41^. By contrast, in studies on the muscarinic M_1_ receptor we showed that M_1_-subtype selective antagonists, including pirenzepine and the closely related ligand telezepine, but not subtype non-selective antagonists, including the prototypic anti-muscarinic drug atropine, also altered receptor quaternary structure at steady-state^27^. Interestingly, in this case pirenzepine increased the proportion of receptor dimer/oligomers. To provide a potential molecular explanation of the selective effects of spiperone and haloperidol compared to eticlopride, nemonapride and clozapine, we exploited a combination of ligand docking studies and molecular dynamics simulations. Based on a study of long time-scale simulation of haloperidol and clozapine in complex with hD_2_R and hD_3_R^23^, we reproduced such dockings, inferred the docking of spiperone and nemonapride and ran 500 ns molecular dynamic simulations. Spiperone and haloperidol share a fluoro-butyrophenone moiety, which is positioned deeply in the TMD bundle and located close to TMD V. Our dynamics simulation with spiperone observed this moiety to reach slightly more deeply into the bottom of the binding site than haloperidol, in proximity to residues Pro5.50, Ile3.40 and Phe6.44. This group of residues are part of the “transmission switch” that provides early stage activation of GPCRs. Unlike spiperone, haloperidol has a chloro-phenyl moiety which protrudes between the external side of TMD II and TMD III, altering the relative distance between these helices. Thomas *et al*.^23^, also considered the mode of binding of clozapine to D_3_R, which unlike haloperidol and spiperone did not promote monomerization of the D_3_R. Here, the mode of binding was very different from that observed for haloperidol as clozapine has a compact chemical scaffold, more akin to the D_3_R-co-crystallized ligand eticlopride. Like clozapine, nemonapride has a more compact scaffold as compared to spiperone or haloperidol. Docking showed it to bind in a more superficial way than the other ligands and we did not observe any significant changes in distances between TMDs. Recently a mutagenesis analysis of TMDs in D_3_R dimers concluded that residues from within TMD IV and TMD V provide a key interface^20^. As the predominant binding pose of spiperone in the receptor results in alterations in this region it may provide a molecular explanation for both destabilization of D_3_R dimers and for the selectivity of this ligand in exerting this action. Interestingly, while our molecular dynamics simulations indicate that binding of spiperone significantly increases the distance between TMD IV and TMD V at the extracellular face of the D_3_R compared to the apo-protein in the absence of a ligand, such an effect was not produced by binding of any other ligand considered in this study. Nontheless, previous mutagenesis studies also highlighted a potential key role for residues near the extracellular face of TMD I and TMD II in the stabilization of the second D_3_R dimeric configuration. Thus it is of importance that our dynamics simulations indicated that binding of spiperone-related haloperidol significantly increases the distance between TMD I and TMD II at the extracellular face of the D_3_R compared to the apo-protein. Although these are inherently predictions, further application of this combination of approaches, linked to both further mutagenesis studies and, indeed, medicinal chemistry, are likely to help validate or repudiate this putative basis of selective ligand regulation of D_3_R quaternary organization. These studies emphasize that chemically different ‘antagonist’ ligands can potentially stabilize distinct, inactive states of a GPCR. Given that it is now widely accepted that different ‘agonist’ ligands can stabilize detectably different states of a GPCR, such outcomes should not be considered surprising and further illustrate that the binding of different ligands to their receptor targets must be considered as producing individual and unique complexes that may alter functional outcomes and the effectiveness of different drugs in a clinical context.

Although studies are limited to approaches like the detection of SDS-resistant ‘dimers’ in immunoblotting studies, Wang *et al*.^49^, have reported increases in the proportion of D_2_-receptor dimers in striatal tissue both in schizophrenia patients and also in an amphetamine-induced sensitized state in a rodent model. There are also reported effects on levels of the D_2_ receptor ‘high agonist affinity’ state in similar situations^50^. Although requiring a great deal of further analysis it is of interest, therefore, that treatment with haloperidol reverses the chronic amphetamine-induced development of the D_2_ receptor ‘high agonist affinity’ state^50^. This supports the clinical relevance of D_2_ receptor oligomerization. Although focus in the above noted studies was on the D_2_-receptor, anti-psychotic agents generally also interact effectively with D_3_R. It would be of interest, therefore, to know if drugs such as eticlopride and clozapine mimic or not the effects of haloperidol in such models.

With increasing attention being paid to differences in the clinical effectiveness of drugs that nontheless interact with the same receptor or suite of molecular targets, it will now be important to examine more closely whether variation in the capacity to alter the quaternary organization of such drug receptors might help to differentiate and classify ligands as regards their functional and therapeutic profiles.

## Experimental Proceedures

### Materials

General laboratory chemicals, as well as poly- *D*-lysine, dopamine, haloperidol, spiperone, (+)-butaclamol, clozapine, sodium butyrate and both anti-tubulin and secondary horseradish peroxidase conjugated antibodies were from Sigma-Aldrich (Poole, UK) or Fisher Scientific (Leicester, UK). Otherwise, DNA restriction endonucleases, calf intestinal alkaline phosphatase, T4 DNA polymerase and T4 ligase were from New England Biolabs (Hitchin, UK). Primers utilized in molecular biology procedures were provided by MWG Operon (Acton, UK). Wizard Plus SV Miniprep kit was from Promega (Southampton, UK). NuPage Novex pre-cast 4–12% Bis-Tris gels and NuPage MOPS SDS running buffer were from Invitrogen (Paisley, UK). QIAfilter Plasmid Maxi Kit, PCR purification kit and QIAquick gel extraction kit were from Qiagen (Crawley, UK). Agarose was from Flowgen Biosciences (Nottingham, UK). The anti-GFP antiserum was generated in-house. Complete protease inhibitors mixture was from Roche Diagnostics (Burgess Hill, UK). ECL reagent was purchased from Pierce (Tattenhall, UK). [^3^H]spiperone and [^35^S]GTPγS were from Perkin Elmer (Chalfont Road, UK). Eticlopride and nemonapride were from Tocris (Bristol, UK). Hank’s Balanced Salt Solution (HBSS) was from Life Technologies (Paisley, UK).

### DNA constructs

Enhanced Green Fluorescent Protein incorporating an A206K mutation to reduce any tendency for the fluorescent protein to homodimerize (mEGFP)^28^ was a gift of Dr K. Herrick-Davis (Albany, New York). In order to localize mEGFP to the plasma membrane a palmitoylation-myristolation (P-M) sequence was added to the amino terminal of the fluorescent protein, as in refs 27 and 42. hD_3_R-mEGFP construct was made by subcloning PCR amplified hD_3_R between the KpnI and SmaI sites of pEGFPN-1 plasmid (Takara Bio Europe/Clontech, Saint-Germain-en-Laye, France). The ‘QuikChange’ method (Stratagene, Agilent Technologies, Santa Clara, CA) was used to introduce the alteration Asp^110^Ala into hD_3_R-mEGFP. Template DNA was digested with DpnI to leave only the newly synthesized mutated plasmid. All the constructs were verified by sequencing.

#### Cell lines

All cells were maintained in a humidified incubator with 95% air and 5% CO_2_ at 37 °C. Parental Flp-In T-REx 293 cells (Invitrogen, Paisley, UK) were maintained in DMEM (high glucose) supplemented with 10% (v/v) fetal calf serum, 100 U.ml^−1^ penicillin, 0.1 mg.ml^−1^ streptomycin, 10 μg.ml^−1^ blasticidin and 100 μg.ml^−1^ zeocin. Flp-In T-REx 293 P-M-mEGFP cells were maintained in DMEM (high glucose) supplemented with 10% (v/v) fetal calf serum, 100 U.ml^−1^ penicillin, 0.1 mg.ml^−1^ streptomycin, 10 μg.ml^−1^ blasticidin and 200 μg.ml^−1^ hygromycin. Flp-In T-REx 293 hD_3_R-mEGFP cells were maintained in DMEM (high glucose) supplemented with 10% (v/v) fetal calf serum, 100 U.ml^−1^ penicillin, 0.1 mg.ml^−1^ streptomycin, 10 μg.ml^−1^ blasticidin and 1 mg·ml^−1^ G418.

#### Stable cell line generation

Inducible Flp-In T-REx 293 stable cell lines able to express P-M mEGFP were generated as described by ref. 41. Flp-In T-REx stable cell lines able to express hD_3_R-mEGFP or hD_3_R-Asp^110^Ala-mEGFP constitutively were generated by transfecting parental Flp-In T-REx cells with hD_3_R-mEGFP or hD_3_R-Asp^110^Ala-mEGFP and antibiotic-resistant clones selected using 1 mg·ml^−1^ G418.

#### mEGFP fluorescence intensity measurement

Cells were grown to 100,000 cells per well in solid black 96-wells plates (Corning) coated with 0.1 mg per ml poly-*D*-lysine. On the day of the experiment, cells were washed with HBSS (3 times on ice). Plates with 100 µl per well HBSS were then read on a PheraStar FS (BMG Labtech, Ortemberg, Germany) fluorescent intensity compatible reader and the emission signal from the mEGFP (520 nm) was recorded after excitation at 485 nm.

#### Cell treatments

For antagonist treatments and sodium butyrate studies cells were incubated with the appropriate concentration of compound or salt for 16 h at 37 °C. For the short term treatment studies cells were treated with 10 µM spiperone for 2 h at 37 °C and then washed six times with medium before being incubated for a further 4 h at 37 °C before confocal images were collected.

#### Cell lysate preparation

Cells were harvested in ice cold phosphate buffered saline (PBS) (120 mM NaCl, 25 mM KCl, 10 mM Na_2_HPO_4_ and 3 mM KH_2_PO_4_, pH7.4) and lysed in lysis buffer (150 mM NaCl, 0.01 mM Na_2_HPO_4_, 2 mM EDTA, 0.5% n-*dodecyl*-ß-D-maltoside (DDM), 5% glycerol and supplemented with ‘Complete’ protease inhibitors mixture) on a rotating wheel for 30 min at 4 °C. Samples were then centrifuged for 15 min at 21000 × g at 4 °C, aliquoted and stored at −20 °C until required.

#### Immunoblotting assays

Cell lysate samples prepared as above were diluted to a final concentration of 2 mg.ml^−1^ in lysis buffer. Samples were prepared by the addition of SDS-PAGE sample buffer and heated to 65 °C for 5 min. 5 μg of protein from each sample was loaded into wells of 4 to 12% BisTris^3^ gels and subjected to SDS-PAGE analysis using NuPAGE MOPS SDS running buffer. After separation, the proteins were electrophoretically transferred onto nitrocellulose membrane, which was then blocked (5% fat-free milk powder in PBS) for 1 h at room temperature on a rotating shaker. The membrane was then incubated with appropriate primary antibody in 5% fat-free milk powder in PBS supplemented with 0.1% Tween 20 (PBS-Tween) overnight at 4 °C on a rotating shaker. Anti-GFP antiserum was diluted 1:10000 and anti-α-tubulin antiserum diluted 1:5000. Subsequently, the membrane was washed (3 × 10 min with PBS-Tween) and then incubated for 1 h with the appropriate secondary antibody (horseradish peroxidase-linked rabbit anti-goat IgG or horseradish peroxidase-linked sheep anti-mouse) diluted 1:10000 in 5% fat-free milk powder in PBS-Tween. After washing (3 × 10 min with PBS-Tween), proteins were detected by enhanced chemiluminescence according to the manufacturer’s instructions.

#### Cell membrane preparation

Cells were harvested in ice cold PBS and pellets of cells frozen at −80 °C, for a minimum of 30 min. These were subsequently thawed and resuspended in ice-cold 10 mM Tris, 0.1 mM EDTA, pH 7.4 (TE buffer) supplemented with Complete protease inhibitors mixture. Cells were homogenized on ice by 40 strokes of a glass-on-Teflon homogenizer followed by centrifugation at 200 × *g* for 10 min at 4 °C to remove unbroken cells and nuclei. The supernatant fraction was transferred to ultracentrifuge tubes and subjected to centrifugation at 90000 × *g* for 45 min at 4 °C. The resulting pellets were resuspended in ice-cold TE buffer and passed through a 25-gauge needle 3 times before being assessed for protein concentration. Membrane preparations were then aliquoted and stored at −80 °C until required.

#### [^3^H]spiperone binding studies on membrane preparations

Both single concentration binding studies and saturation binding curves were established by the addition of 5 µg of membrane protein to assay buffer (20 mM HEPES, 6 mM MgCl_2_, 1 mM EDTA, 1 mM EGTA, 40 μM ascorbic acid) containing either a single, near saturating, concentration (5 nM) or varying concentrations (0.019–14 nM) of [^3^H]spiperone in 200 µl final volume. Non-specific binding was determined by the addition of 10 μM (+)-butaclamol. Reactions were incubated for 2 h at 30 °C and terminated by rapid vacuum filtration though GF/C glass fiber filters followed by three washes with ice-cold PBS. The level of radioactivity associated with the filters was quantified using a TriCarb 2810 Tr scintillation counter. Competition binding assays were carried out in a similar way, but with a constant concentration of [^3^H]spiperone (1 nM) and the addition of a range of concentrations of ligands of interest. Data were analysed using Graphpad Prism 5.03 (Graphpad Inc, CA, USA).

#### [^3^H]spiperone binding studies on intact cells

Cells were grown to 100000 cells per well in solid white 96-wells plates (Corning) coated with 0.1 mg per ml poly-*D*-lysine. On the day of the experiment cells were washed with HBSS (3 times on ice) and 5 nM [^3^H]spiperone was added to appropriate wells containing or not 10 μM (+)-butaclamol to determine non-specific binding in 100 µl final volume. Plates were incubated for 4 h at 4 °C and the reactions were terminated by removal of the binding mixture followed by washing with 4 × 200 μl per well ice-cold HBSS. One hundred microliters per well microscint 20 (Perkin Elmer Life Sciences) was added and the plates were sealed before overnight incubation at room temperature on a rapidly shaking platform. Bound ligand was determined using a Packard Topcount NXT (Perkin Elmer Life Sciences). Using the specific binding per well and number of cells per well, receptor copy per cell was determined.

#### [^3^H]spiperone dissociation studies

Were carried out according to the method of the “infinite dilution” achieved by abundantly washing cells and resuspending them in ligand-free buffer. In details, cells were plated at 70,000 cells per well in 96-well plates 24 h before the assay. On the day of the experiment, cells were washed with Hanks’ balanced salt solution (HBSS) 3 times and then 0.5 nM [^3^H]spiperone was added to each well in the absence or presence of 10 μM (+)-butaclamol to determine non-specific binding, in 100 µl final volume. Plates were incubated for 1 h at 37 °C and the reactions was terminated by five washes with HBSS. To monitor radioligand dissociation 100 µl per well HBSS was added to each well and this was replaced with 100 µl per well Microscint 20 at various times.

#### *[*^*35*^*S] GTP***γ***S Binding Assays*

Cell membranes (10 μg) were incubated in 900 μl of buffer (20 mM HEPES, 100 mM NaCl, 6 mM MgCl_2_, 40 μM ascorbic acid, pH 7.4) containing 1 μM GDP, 10 μM dopamine (where indicated) and receptor saturating concentrations of the appropriate antagonist. All experiments were performed in triplicate. The reaction was initiated by the addition of cell membranes and incubated at 30 °C for 30 min. A 100-μl volume of [^35^S]GTPγS (0.1 nM final concentration) was then added, and the incubation was continued for a further 30 min. The reaction was terminated by rapid filtration with a Brandel cell harvester and three 4-ml washes with ice-cold phosphate-buffered saline. Radioactivity was determined as described for saturation analysis.

#### Spatial Intensity Distribution Analysis (SpIDA)

Was carried out essentially as described in refs 27 and 41. All RoI measurements were selected from the basolateral membrane surface. Unbiased RoI selection was evaluated for each experimental group by normalizing the quantified mean fluorescence intensity of the whole background corrected image to the mean fluorescence intensity measured from each RoI analysed within that image. The fraction range varied from 0.93 ± 0.02 (spiperone treated sample) to 0.80 ± 0.06 (nemonapride treated sample) and in no case was the fraction values significantly different when compared with those obtain for the untreated sample indicating that the total receptor population had been analyzed competently and no unbiased selection had occurred. MEU values for hD_3_R-mEGFP were measured by normalizing their quantified QB values to average QB values measured from the P-M-mEGFP construct using exactly the same laser power as used to excite the hD_3_R-mEGFP construct. To distinguish between monomeric and dimeric/oligomeric hD_3_R-mEGFP species, P-M-mEGFP MEU occurrence/frequency x-y graphs (MEU bin size = 0.1) were plotted for each MEU value measured during excitation with laser power set to 2 or 6%. Such plots revealed a symmetrical distribution of the values and Graphpad Prism normality tests indicated the distributions were Gaussian (see Results and Statistical analyses). The data from each frequency x-y plot measured using 2 or 6% laser power were combined as this range of excitation settings was required to illuminate the hD_3_R-mEGFP optimally at differing expression levels without erroneous detector pixel saturation occurring. From this combined plot, an MEU value of 1.406, (which represented 95.44% of the data set, falling within the mean + 2 standard deviations), was set as a threshold to distinguish between monomeric and larger complexes in studies where individual MEU values exceeded 1.406.

#### Calculation of receptor density at the cell surface by SpIDA

SpIDA software also reports the mean fluorescent intensity for each RoI analyzed. The number of hD_3_R-mEGFP or P-M-mEGFP molecules per μm^2^ (density) was measured by dividing the mean fluorescent intensity value by the quantified monomeric QB value.

#### Calculation of Flp-In T-REx 293 cell surface area

3D stacks of Flp-In T-REx 293 cell expressing the mEGFP tagged 5-HT_2C_ receptor at the membrane surface^41^ were recorded on a Zeiss Exciter confocal using a 63 x oil immersion Plan Apochromat objective lens using a step z size of 0.26 microns. The 3D stacks were loaded into the software program Autodeblur/Autovisualise, (version 9.3.6, Autoquant Imaging, Watervliet, NY), and receptor fluorescence within the 3D stack for each individual image was segmented using the adaptive thresholding tool to resolve mEGFP-tagged receptor fluorescence from background autofluorescence. The total surface area was then subsequently quantified using the surface area volume tool.

#### Fluorescence recovery after photobleaching (FRAP)

Image Acquisition: Fluorescence recovery after photobleaching (FRAP) experiments were performed using a Zeiss 510 Exciter laser scanning confocal equipped with a 63x/1.4 NA Plan Apochromat oil-immersion objective. The 488 nm argon laser line was used to sequentially excite mEGFP and the image format size was set to 256 × 256 to speed up sequential 2.5 s time lapse image collection. To minimise mEGFP fluorescence photobleaching the 488 nm laser line was set to 4% for acquisition of the pre-bleach and post-bleach recovery images. Bleaching was performed on the basolateral membrane of cells by setting the laser power to 100% and the RoI bleached spot size was set to 3 µm in diameter. 5 pre-bleach images and 44 recovery images were acquired to quantify the degree of fluorescence recovery after the laser RoI photobleaching event. FRAP analysis: Time lapse stack images generated from each experimental group were imported into Metamorph software, (version 7.8), and auto-aligned to correct for x-y drift. For each experimental image stack set, a circular RoI was drawn within the observed bleached area, (RoI Fs), and the average fluorescence intensity within the RoI Fs was quantified for each individual image within the stack to quantify the average level of fluorescence before and after photobleaching. The degree of background autofluorescence was quantified similarly by drawing an RoI in an area where no cell fluorescence was expressed, (RoI Fb). To monitor the native photobleaching rate (r), for each FRAP experiment, a control RoI was drawn on a non-bleached cell within the time lapse image series. The photobleaching rate (r) was then quantified by comparing the average fluorescence intensity of the control RoI at time zero, (Fc0), with the corresponding measured average fluorescence intensity at the end of the experiment, (Fc), e.g. r = Fc/Fc0. The average fluorescence intensity of the Fs RoI for each stack image was corrected for the degree of native photobleaching and background autofluorescence for each FRAP experiment as follows: F = (RoI Fs − RoI Fb)/r. The F data was the normalized, (0–100%), and the recovery half-life and mobile fraction were quantified using the Graphpad Prism software one-phase exponential equation.

### Computational methods

A modification of the crystal structure of hD_3_R, in complex with eticlopride, (PDB code: 3PBL)^22^ was used to build all the D_3_R–ligand complexes or the apo-protein models. As the N-terminal of TMD I in hD_3_R crystals is about 2 helix turns shorter than all other class A GPCR structures released to date, Modeller 9v8^51^ was used to model a TMD I as long as observed in the turkey ß_1_-adrenoceptor^52^. Haloperidol and clozapine where docked as by Thomas *et al*.^23^, while nemonapride and spiperone were docked into the modified hD_3_R crystal structure using the Autodock Vina tool^53^. All docking poses were visually inspected and two more frequent docking possibilities were observed. In the case of nemonapride they were similar and two dynamics simulation converged on the first poses, while in the case of spiperone in the first pose the fluoro-butyrophenone moiety of spiperone was deeply buried in the binding pocket, in a similar conformation to the one described by Thomas *et al*.^23^ for the hD_3_R-haloperidol complex (which shares this chemical feature with spiperone) whilst in the second the fluoro-butyrophenone moiety of spiperone faces the extracellular side of TMD 1. As both these conformations have been reported in the literature we employed molecular dynamics simulations to assess which was more stable. This showed the only conformation stable in a 500 ns molecular dynamics simulations was that akin to that reported by Thomas *et al*. for haloperidol^23^. On this basis we selected this as the docking pose. Ligand-hD_3_R complexes were embedded in a lipid bilayer (187 molecules of POPC) with explicit solvent (14067 water molecules) and counter-ions (58 Na^+^ and 67 Cl^−^). Model systems were energy minimized and subsequently subjected to a 27 ns molecular dynamics equilibration, with incremental positional restraints on non-hydrogen atoms, main chain, loop free and the Cα atoms of the receptor, to remove possible voids present in protein/lipids or protein/water interfaces. These restraints were released and 500 ns MD trajectories were produced at constant pressure and temperature, using the particle mesh Ewald method to evaluate electrostatic interactions with the GROMACS software^54^ v5.1, the AMBER99SB force field for the amino acids, and Berger parameters for POPC lipids, using the protocol previously described^55^. Analyses of the trajectories were made using VMD v1.9.3^56^.

### Statistical analyses

Variation in receptor number among the three different clones considered or produce by treatment with ligands was assessed by 1-way ANOVA with the use of Bonferroni’s or Dunnett’s test for multiple comparisons as appropriate. Variation in receptor number produced by treatment with sodium butyrate was assessed by Student’s *t* test. Variation in mean of QB was assessed by Student’s *t* test or 1-way ANOVA with the use of Dunnett’s test for multiple comparisons as appropriate. Normality distributions of recovered QB values defined as MEUs were assessed by of D’Agostino and Pearson (at p > 0.05) and by skewness and Kurtosis assessments. Distributions that failed the normality assessment (at p < 0.05) were considered to be non-Gaussian. Variation in [^3^H]spiperone binding was assessed by 1-way ANOVA with the use of Bonferroni’s or Dunnett’s test for multiple comparisons as appropriate. Variation in [^35^S]GTPγS binding was assessed by 1-way ANOVA with the use of Dunnett’s test for multiple comparisons. Variation in the half-time and mobile fraction in FRAP experiments were assessed by 1-way ANOVA with the use of Bonferroni’s for multiple comparisons. Variation in the distances between residues within helices in molecular dynamics simulations were assessed by 1-way ANOVA with the use of Tukey’s for multiple comparisons.

## Electronic supplementary material

Supplementary data
