# Supplementary material for: A Molecular Basis for Selective Antagonist Destabilization of Dopamine D3 Receptor Quaternary Organization
Source: Sci Rep. 2017 May 18;7:2134. doi: 10.1038/s41598-017-02249-3 (PMC5437050; doi:10.1038/s41598-017-02249-3)
Supplement: Supplementary file 1 — Supplementary data [file 41598_2017_2249_MOESM1_ESM.pdf]

A Molecular Basis for Selective Antagonist Destabilization of Dopamine D_3 Receptor Quaternary Organization

Sara Marsango[1*], Gianluigi Caltabiano[2], Mireia Jiménez-Rosés[2], Mark J. Millan[3], John D. Pediani[1], Richard J. Ward[1] and Graeme Milligan[1*]

[1]Centre for Translational Pharmacology, Institute of Molecular, Cell and Systems Biology, College of Medical, Veterinary and Life Sciences, University of Glasgow, Glasgow G12 8QQ

Scotland, U.K.

[2]Laboratori de Medicina Computacional, Unitat de Bioestadística, Facultat de Medicina, Universitat Autònoma de Barcelona, 08193 Bellaterra, Spain

[3]Institut de Recherches Servier, Unité de Recherche et découverte en Neurosciences, 125 Chemin de Ronde, Croissy sur Seine, France 78290

Address correspondence to Graeme Milligan (Graeme.Milligan@glasgow.ac.uk, tel 44 141 330 5557, or Sara Marsango (Sara.Marsango@glasgow.ac.uk)

Supplemental Material

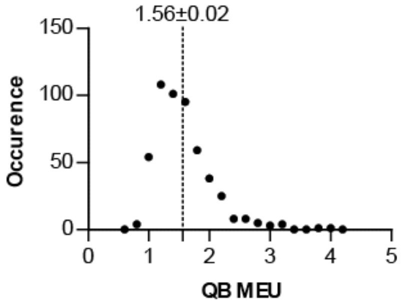

Supplemental Figure 1. The distribution of the full QB MEU data set across all experiments reported that were generated from hD$_3$R-mEGFP expressing cells of clone 2 (bin size, 0.2 MEU) is shown. These are distributed in a Gaussian fashion, 1.56 ± 0.02 (mean ± S.E.M., n=515).

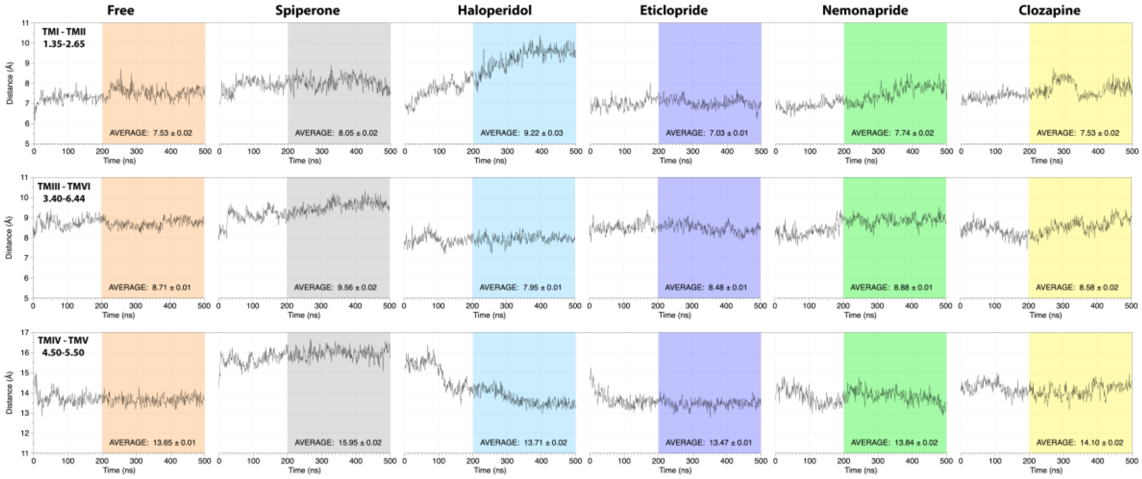

Supplemental Figure 2. Molecular dynamics simulations of the hD$_3$R apo-protein, or hD$_3$R bound to spiperone, haloperidol, eticlopride, nemonapride or clozapine were run for 500 ns. Each point represents average of 3 independent replicas of the distances between C-α atoms of residues located at positions 1.35 and 2.65 (**top panels**), 3.40 and 6.44 (**middle panels**) and 4.50 and 5.50 (**lower panels**). Average values reported are of the last 300 ns ± S.E.M.

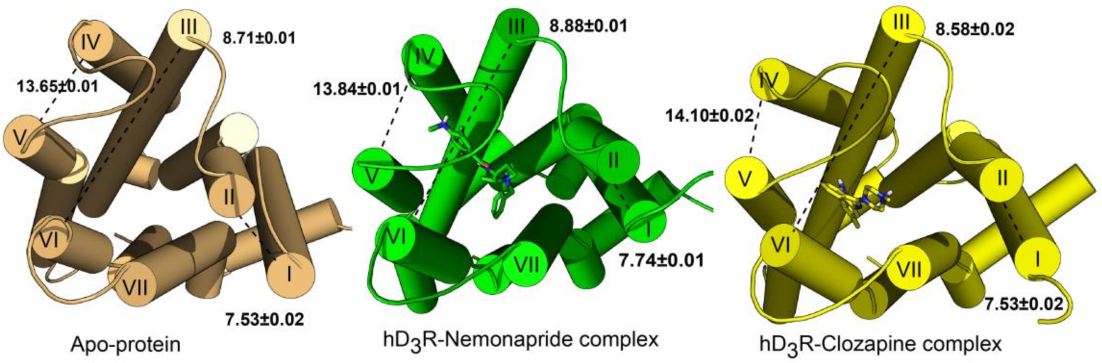

Supplemental Figure 3. Representation of the tertiary structure of the receptor at the end of molecular dynamic simulations. The distances between residues within helices (calculated as average distance, n=3, between pairs of C-α atoms: 1.35 and 2.65, 3.40 and 6.44, 4.50 and 5.50) are shown.
